# Three-dimensional assessment of the skeletal characteristics accompanying unilateral maxillary canine impaction: a retrospective cone-beam computed tomography study

**DOI:** 10.1186/s12903-024-04974-4

**Published:** 2024-10-19

**Authors:** Farah Y. Eid, Sherifa I. Ghaleb, Fatma F. Badr, Eiman S. Marzouk

**Affiliations:** 1 Department of Orthodontics, Faculty of Dentistry, Alexandria University, Champolion street, Alexandria, Egypt; 2 Oral Diagnostic Sciences Department, Faculty of Dentistry, King Abdulaziz University, Jeddah, Saudi Arabia

**Keywords:** Cone-beam computed tomography, Unilateral impaction, Bone density, Bone microstructure, Fractal dimension, Bone volume, Palatal volume

## Abstract

**Background:**

Environmental and genetic factors associated with canine impaction have been extensively researched, whereas the bone characteristics in the impaction area have not been thoroughly studied. Accordingly, the objective of this investigation was to provide a skeletal assessment in terms of bone density, bone microstructure, bone volume, and palatal volume in subjects with unilaterally impacted maxillary canines.

**Methods:**

A retrospective design has been employed to address the aim of this study, where the initial pre-treatment cone-beam computed tomography (CBCT) scans of 30 patients with unilateral maxillary canine impaction were assessed. The obtained patients’ data were equally divided according to the location of the impaction into 2 groups, one with buccally impacted canines, and another with palatal impactions, with the contra-lateral sides in both groups serving as the controls. Skeletal measurements such as bone density (BD), bone microstructure in terms of fractal dimension (FD), maxillary bone volume (MBV), and palatal volume (PV) were evaluated from the acquired CBCTs in both groups and compared to the controls.

**Results:**

With buccal impactions, significantly greater BD and FD have been reported (*p* < 0.001), whereas non-significant differences were found regarding the PV when compared with controls (*p* = 0.56). MBV was significantly greater on the non-impaction side in comparison with buccal impaction sides (*p* < 0.001). For palatal impactions: BD, FD, and MBV were significantly greater on the impaction sides (*p* < 0.001), and conversely with PV which has been reported to be significantly greater on the non-impaction sides (*p* < 0.001).

**Conclusions:**

As per the obtained results, buccally impacted canines are associated with greater BD and FD, and less MBV, whereas palatally impacted canines are accompanied with greater BD, FD, and MBV, in addition to less PV, when both conditions are compared with the non-impaction sides.

## Background

Maxillary canines have been reported to be the second most frequently impacted teeth after third molars, and it has been shown to affect approximately 1–3% of the general population [1, 2], with unilateral impactions being more common than bilateral ones [3].

With new developments in medical imaging, methods for locating impacted canines have been substantially improved. In consequence, cone-beam computed tomography (CBCT) was reported to offer three-dimensional multiplanar images and details on dentofacial structures, making it much more preferable to traditional radiography [4, 5].

One of the recent applications of CBCT is investigating the trabecular characteristics of the maxillary bone. Qualitative skeletal assessment involves the measurement of bone density (BD) [6] and microstructure [7]. Even though BD is commonly measured in grey scale values (GSV), it has been reported that these measurement units might not be reliable [8], and that the values might vary according to the employed machines and the sites localized [9, 10]. Consequently, bone microstructure has been proposed as an alternative approach for the assessment of bone quality, and its evaluation is usually in terms of trabecular number and fractal dimension (FD) analysis [11]. Furthermore, studies have reported that there is an existent correlation between the FD values computed form CBCTs using ImageJ processing software, and the BD as well as the trabecular patterning [12–14]. Considering the irregular character of the trabecular bone, assessment of FD could be a more accurate evaluation method in comparison with the traditional BD analysis using GSV [14].

Determining alterations in BD between the impaction and the non-impaction sides provides a better comprehension regarding the impaction etiology, and helps in estimating the difficulty of the disimpaction procedure [15]. Nevertheless, diverse reports have been documented regarding the influence of BD and its correlation with canine impactions. It is still rather perplexing whether elevated BD values could be a contributing factor in the etiology behind canine impaction, or if it is to be considered a consequence of the impaction itself.

From a clinical perspective, BD strongly influences orthodontic treatment duration [16]. Moreover, FD acquired from dental radiography is considered a useful predictor of the initial stability of dental implants [17]. Fractal analysis can also assist the practitioner in the quantitative evaluation of radiographic images as a screening tool to predict the difficulty of orthodontic treatment, planning surgical interventions if necessary, and anticipating potential complications. This information allows for a more personalized treatment planning process, thereby enhancing treatment outcomes and patient satisfaction [18].

Bone volume (BV) has been related to quantitative assessment of bone. However, similar to the case with BD, former studies have not considered maxillary BV as a possible etiological factor in canine impaction. The only study evaluating BV in conjunction with maxillary canine impaction was that by Al-Tawachi et al. [19], where an increase in BV was reported with palatally displaced canines, and opposingly, a BV reduction has been observed with the buccally displaced ones.

On another note, assessment of the palatal volume (PV) is one of the helpful factors in decision-making, as it provides a rational representation of the palatal conditions [20]. Measurement of PV has always been considered quite complicated, where dental models were employed at first [21], followed by three-dimensional (3D) digital models [22]. However, image noise and obscure borders have been reported as common defects encountered with these 3D images, all of which CBCT scans were reported to overcome in volumetric analysis [23].

In conclusion, the aim of our study was to perform a comprehensive skeletal assessment in unilateral canine impaction cases, whether buccally or palatally positioned, including bone density, bone microstructure in terms of fractal dimension, bone volume, and finally palatal volume. The null hypothesis is that there are no significant differences regarding the assessed skeletal factors in patients exhibiting unilateral canine impaction (buccal or palatal), in comparison with the contra-lateral sides with fully erupted canines.

## Methods

### Patient selection

A retrospective design was employed to address the aim of the study. Ethical approval was attained from the Institutional Review Board of the Faculty of Dentistry, Alexandria University, Alexandria, Egypt (IORG:0008839, Ethics Committee number 0771-09/2023. The pre-treatment orthodontic records of patients treated at the Department of Orthodontics, Faculty of Dentistry, Alexandria University have been collected and screened for eligibility by the principal investigator to secure the recommended sample size.

The sample size was calculated assuming 80% study power and 5% alpha error. Arvind et al. [7] reported mean ± SD bone fractal dimension = 1.47 ± 0.24 in the impaction side, and 1.21 ± 0.09 in the non-impaction side, with a mean ± SD difference = 0.26 ± 0.17, and 95% confidence interval= -0.30, -0.11. The minimum required sample size was calculated to be 15 patients (30 sides). The total required sample size = number of groups × number per group = 2 × 15 = 30 patients (60 sides) [24]. Sample size estimation was carried out using MedCalc Statistical Software version 19.0.5 (MedCalc Software bvba, Ostend, Belgium; https://www.medcalc.org; 2019).

Inclusion criteria for records’ selection included: (1) Age ranging from 15 to 30 years. (2) The presence of a pre-treatment CBCT scan showing unilateral canine impaction, with good image quality. (3) Buccal or palatal classification of the impaction was based on the root of the adjacent lateral incisor, and its relation to the tip of the maxillary canine crown, whether it was buccal or palatal to the lateral incisor root [25]. (4) Presence of fully erupted contra-lateral canines. (5) No former orthodontic treatment. (6) Presence of a full set of permanent dentition with/without the third molars. While the exclusion criteria included: (1) Syndromic patients, with systemic diseases related to bone health. (2) Presence of pathological conditions, such as cysts, dentoalveolar trauma, craniofacial malformations, and multiple teeth impactions. (3) Presence of aggressive or progressive periodontitis.

Diagnosis of buccal or palatal unilateral maxillary canine impaction was performed initially by one researcher (F.E.) based on the acquired pre-treatment CBCT scans and classified as stated in the inclusion criteria [25]. The same and another investigator (S.G.) re-examined 10 randomly selected CBCTs, two weeks following the initial evaluation, and a 100% agreement was confirmed between both researchers regarding the location of the canine impaction [26].

### Grouping

The selected records were divided into two groups based on the position of the unilateral maxillary canine impaction: Buccal Unilateral Maxillary Canine Impaction (BUMCI), and Palatal Unilateral Maxillary Canine Impaction (PUMCI). Moreover, in each of the two study groups, the contra-lateral sides served as the control group, in the split-mouth design.

### Outcomes

The entire sample of CBCT volumes was acquired using the same CBCT machine (J. Morita R100 Cone beam 3D Imaging System; MFG Corp., Kyoto, Japan). The Field of View (FOV) of the scan was 100 × 50 mm (Width × Height), centered on the maxilla. Volume reconstructions were carried out with a 0.160 mm isometric voxel size. Additionally, the voltage of the tube was 90 kVp and 8 mA, with a 20 seconds’ exposure time.

The following measurements were performed, and each side of the maxillary arch was individually assessed:

#### Bone density (BD)

**Fig. 1:**
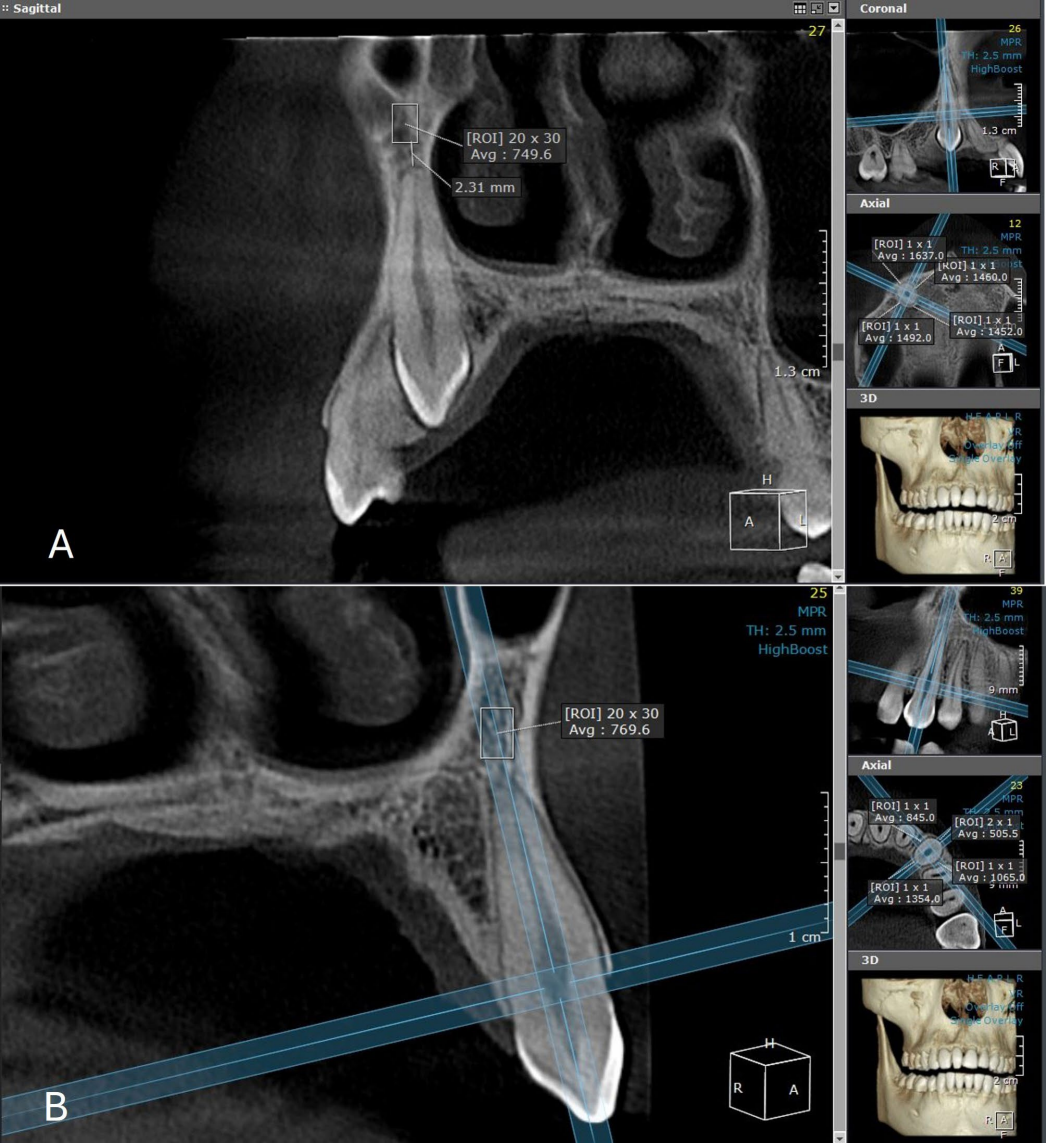
Assessment of BD with a cross-sectional slice taken approximately 3 mm above the maxillary canine root apex (3DModule, OnDemand3D™ App software). **A**: Impaction side. **B**: Non-impaction side

Using the 3D Module on the OnDemand3D™ App software (Cybermed Inc., Seoul, Korea), a cross-sectional slice through the long axis of the canine root has been selected, with a slice thickness of 2.5 mm. A rectangular region of interest (ROI) size of 20 × 30 mm has been determined at a level approximately 3 mm above the maxillary canine root apex, and BD was calculated on both the impaction and the non-impaction sides in the two groups using GSV **(**Fig. 1**)**. The ROI was chosen to be 3 mm above the canine root apex to standardize measurements across patients and minimize variability due to anatomical differences in periodontal ligament space and lamina dura. This location is considered stable and representative of bone quality around the impacted tooth.

#### Fractal Dimension (FD)

**Fig. 2:**
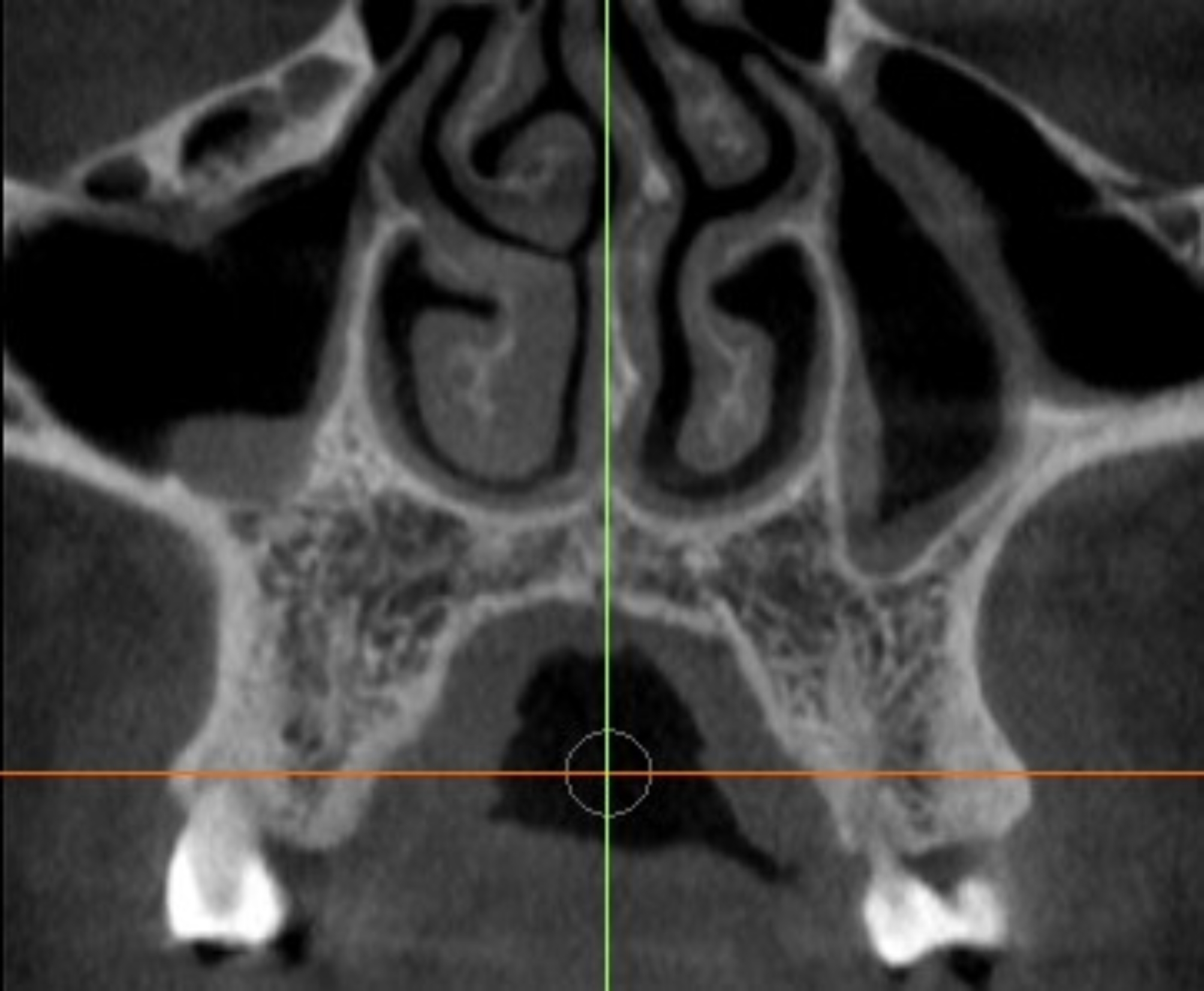
An axial section of trabecular bone located interproximal to the first and second premolars was selected for FD analysis (Anatomage Inc., Santa Clara, CS, US)

The DICOM files were opened using InVivoDental-Intel Application version 6.0.2 (Anatomage Inc., Santa Clara, CA, US). The ROI was selected between the first and second premolars, on both the impaction and non-impaction sides of the selected scans **(**Fig. 2**)** [6]. The selected ROIs were carefully examined to ensure the absence of dental landmarks, nerve canals, and cortical bone. These images were then saved as JPEG files [7]. Using Microsoft Office Picture Manager (Microsoft Corp., Redmond, WA), the selected images were cropped into a 64 × 64-pixel image for further analysis [27].

**Fig. 3:**
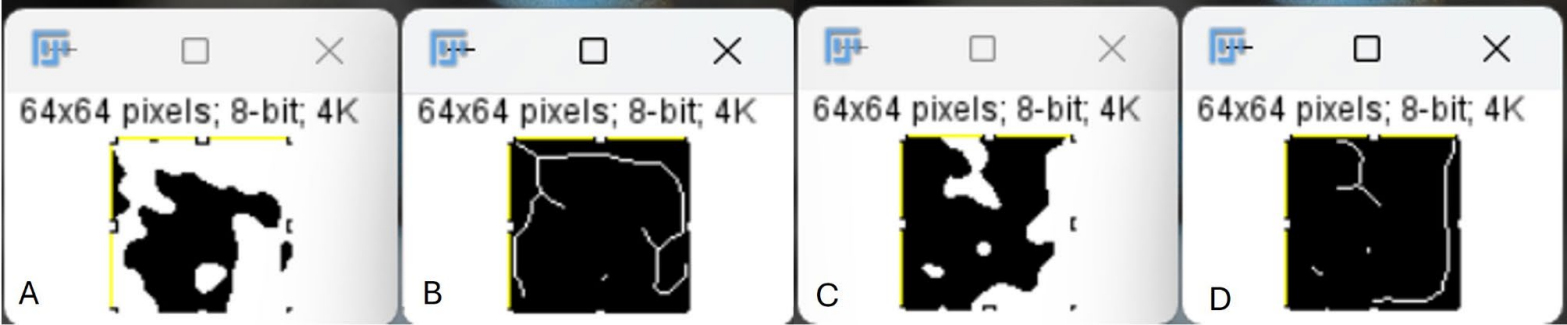
An example of the final skeletonization step prior to running the FD analysis on the impaction side **(A**,** B)** and non-impaction side **(C**,** D)**, and how it transforms the trabecular pattern

Each cropped image was imported to Image J (Fiji) [28] to run the FD analysis steps. The processing steps for the cropped images were replicated from Saberi et al. [29] to ensure accurate representation of the cancellous bone architecture. First, Gaussian blur filter was applied to eliminate brightness variations. Then, subtraction, addition and conversion to a binary image were done. To better visualize cancellous bone trabeculations, erosion and dilation were done to the binary image. Furthermore, skeletonization was performed **(**Fig. 3**)**. Finally, the FracLac plugin was used applying the box counting method to run the fractal analysis.

#### Maxillary Bone volume (MBV)

**Fig. 4:**
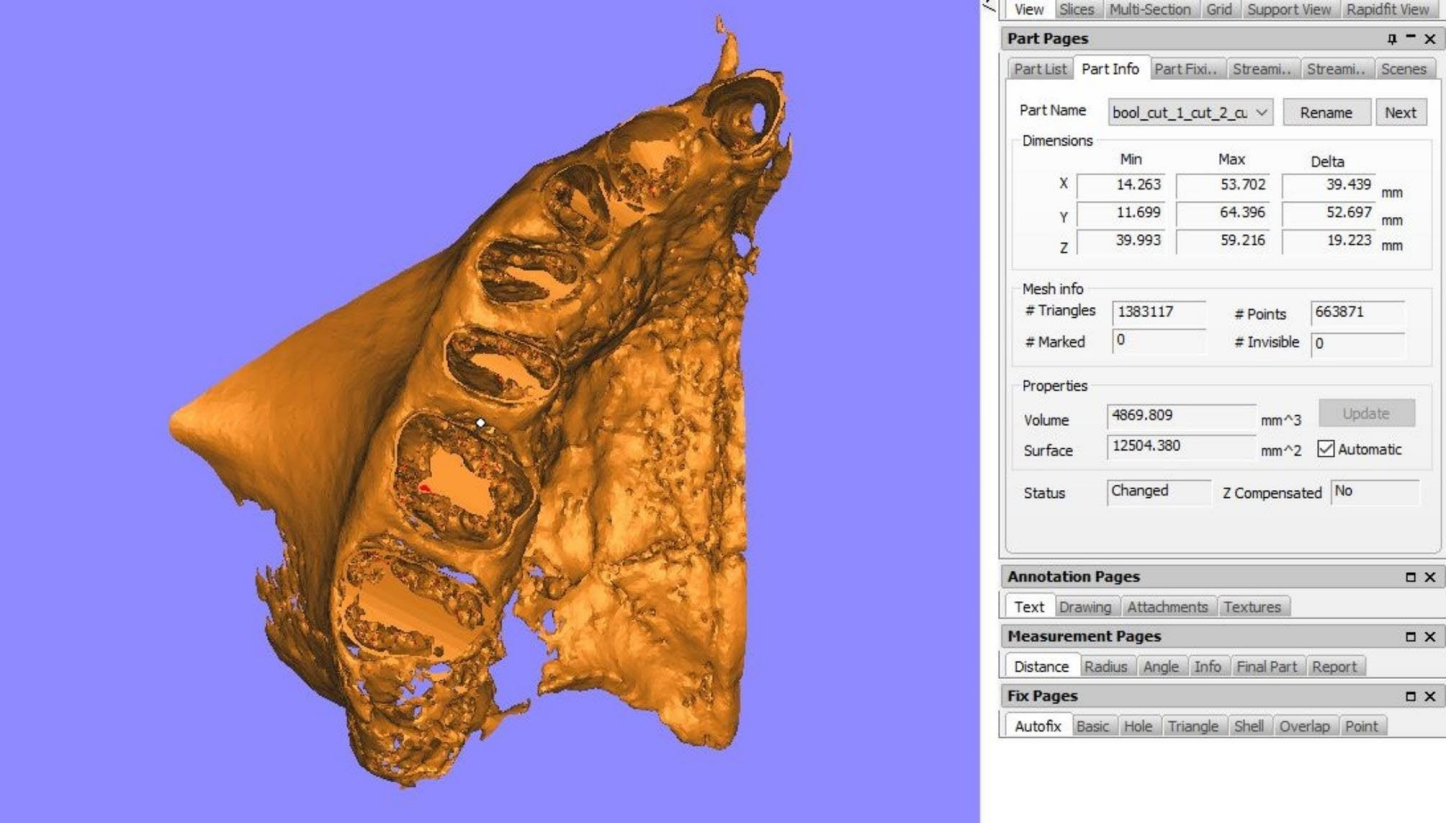
Measurement of MBV on one side of the maxillary arch, after performing a Boolean operation where the volume of the teeth was subtracted from the overall volume (Materialise Magics 3D Print Suite software)

MBV represents the total bone volume surrounding the teeth on the side of interest, and accordingly, the maxilla was split into two segments from the mid-palatal suture. Using the In2Guide Module on the OnDemand3D^™^ App software, the threshold was adjusted twice in each half of the maxilla, once for the teeth alone, and another time for the teeth and the bone together, and the threshold was set individually for each case. The two resultant exported 3D Model Standard Tessellation Language (STL) files from each scan, were subsequently imported into Materialise Magics 3D Print Suite software (Materialise, Belgium), where the maxillary arch was segmented with the following reference points: the anterior nasal spine (ANS) as the anterior limit, the zygomaticomaxillary suture as the posterior limit, the alveolar ridge of the alveolar bone as the lower limit, and finally, the mid-palatal suture as the medial limit. A Boolean operation was then carried out for each maxillary segment, where the volume of the teeth was subtracted from the overall volume of the cut area to determine the bone volume solely. This calculation was automatically performed by the employed software in mm³ **(**Fig. 4**)**.

#### Palatal volume (PV)

**Fig. 5:**
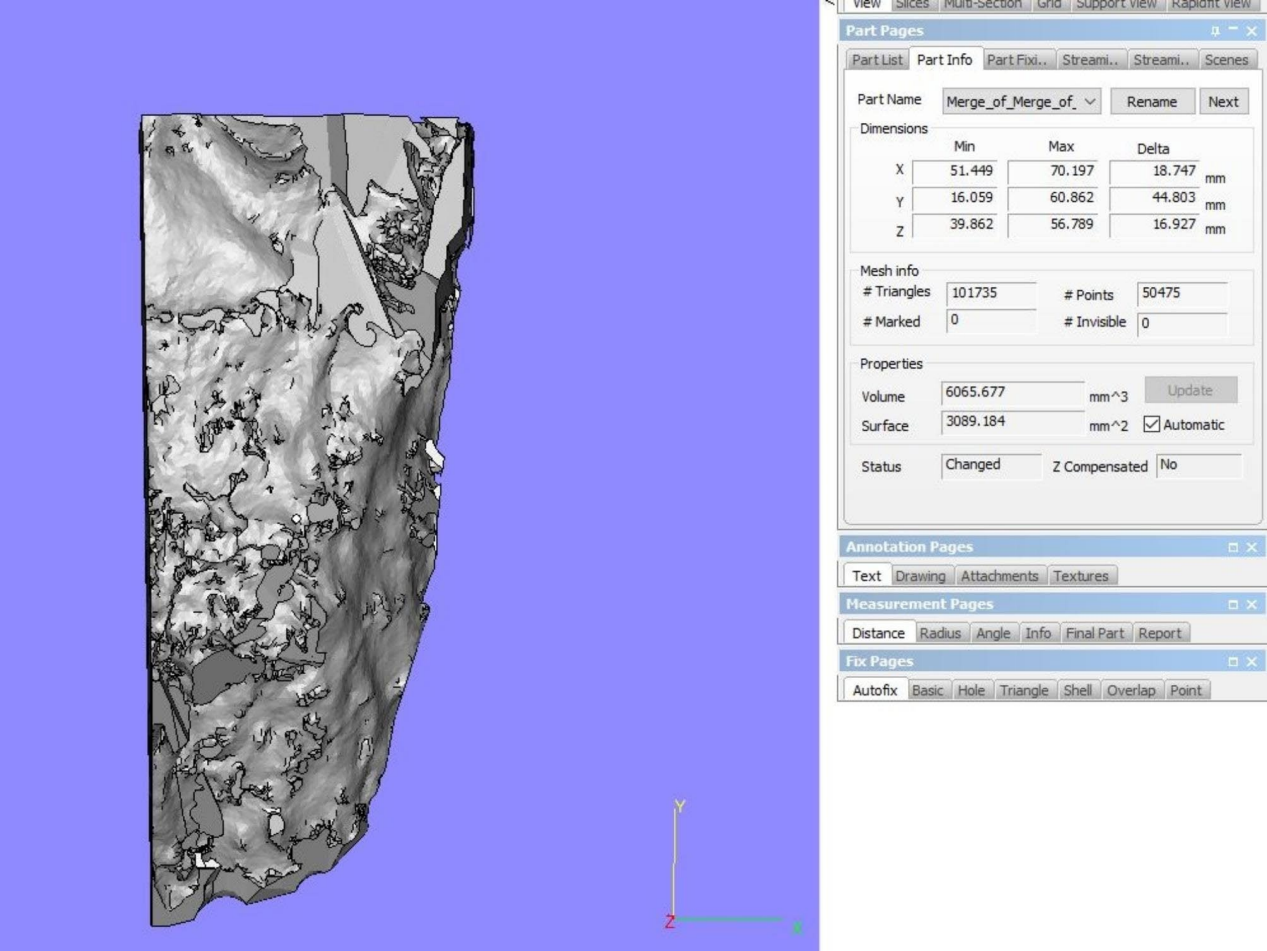
Calculation of PV on one side of the maxillary arch using a palatal object created with anterior, posterior, upper, and lower borders, followed by a Boolean operation removing the respective volumes of the maxillary teeth and bone (Materialise Magics 3D Print Suite software)

The 3D Model STL files generated using the In2Guide of the OnDemand software, that have been subsequently exported into Materialise Magics 3D Print Suite software after threshold adjustment for measurement of MV, have been also employed in the evaluation of the PV, which refers to the volumetric space palatal to the teeth. Using the Materialise Magics software, an object was created to fill the palatal space within the set area of interest. The upper border was the deepest point on the palatal vault. The lower border was represented by a horizontal line drawn from the cemento-enamel junction (CEJ) of the central incisor that extends posteriorly parallel to the horizon. The anterior border was a line joining the CEJ of the central incisor and the deepest point on the palatal vault, whereas the posterior border was delineated by a line that drops perpendicular to the lower border [20]. The created palatal object was then divided at the mid-palatal suture and a Boolean operation was carried out to remove the maxillary teeth and bone in each half, and subsequently calculate the volume of the generated palatal object representing the palatal volume on each side in mm³ **(**Fig. 5**)**.

### Statistical analysis

Normality was tested for the included variables using descriptive statistics, plots (Q-Q plots and histograms), and normality tests. All the data showed normal distribution, so means and standard deviation (SD) were calculated, and parametric tests were used. Comparisons between buccal and palatal impactions were performed using independent samples t-test, while comparisons between impaction and non-impaction sides were carried out using paired samples t-test, with calculation of mean differences and 95% confidence intervals (CI)s. The significance level was set at p-value < 0.05. Data were analyzed using IBM SPSS for Windows (Version 26.0).

## Results

Baseline characteristics of the recruited sample regarding both age and gender are displayed in Table 1, with no significant differences shown between the subjects in both study groups regarding the assessed parameters (*p* > 0.05).

**Table 1:** Demographic characteristics of the two study groups

		BUMCI	PUMCI	*P* value
Age	Mean (SD)	19.40 (5.42)	20.00 (4.44)	0.74 ***a***
Gender: n (%)	Male	7 (46.7%)	8 (53.3%)	0.72 ***b***
	Female	8 (53.3%)	7 (46.7%)	

**a** Chi-square test was used

**b** Independent samples t-test was used

### Intra-examiner and inter-examiner reliability

Study measurements’ calibration was carried out for two assessors, therefore, both intra- and inter-examiner reliability were evaluated, and Intraclass Correlation Coefficient (ICC) [30] was calculated to be 0.825 to 0.982, indicating good to excellent agreement between examiners and across time.

### BUMCI

Values of the measured variables (BD, FD, MBV, and PV) in the BUMCI group on both the impaction and non-impactions sides are presented in Table 2. Outcomes’ assessment reported statistically greater BD on the impaction sides in comparison to the contra-lateral sides with respective values of 672.25 GSV ± 82.28, and 573.26 GSV ± 78.84. Moreover, FD was significantly greater on the impaction sides (1.11 ± 0.04), in contrast to the non-impaction sides (0.89 ± 0.10). For the MBV, statistically greater bone volume values were calculated on the non-impaction sides when compared to the buccally impacted canines’ sides (6873.01 mm³ ± 1401.09, and 5714.74 mm³ ± 1432.29, respectively). Finally for the PV, non-significant differences have been shown between both the tested sides (*p* = 0.56).

**Table 2:** Comparison of different parameters between impaction and non-impaction sides in the BUMCI group

	Impaction side	Non-impaction side	Difference (95% CI)	*P* value
	Mean (SD)			
BD (GSV)	672.25 (82.28)	573.26 (78.84)	98.99 (92.36, 105.61)	< 0.001*
FD	1.11 (0.04)	0.89 (0.10)	0.22 (0.17, 0.27)	< 0.001*
MBV (mm³)	5714.74 (1432.29)	6873.01 (1401.09)	-1158.27 (-142.09, -896.45)	< 0.001*
PV (mm³)	7767.62 (1410.84)	7819.92 (1414.57)	-52.30 (-242.30, 137.70)	0.56

SD: Standard Deviation, CI: Confidence Interval

Paired samples t-test was used

*Statistically significant at p-value < 0.05

### PUMCI

Outcomes of all tested variables in the PUMCI group are displayed in Table 3. Significantly greater BD has been calculated on the impaction sides (806.45 GSV ± 43.40), in contrast to the non-impaction sides (664.16 GSV ± 42.86). Significantly higher FDs have been observed also on the palatal impaction sides (1.21 ± 0.07) when compared to the other non-impaction sides (0.91 ± 0.10). Following a similar pattern was the MBV, with significantly greater values being recorded on the impaction sides (6688.29 mm³ ± 906.26), as opposed to those on the contra-lateral sides (5303.08 mm³ ± 1312.89). Opposingly, PV was significantly less on the sides of palatally impacted canines in comparison to the non-impaction sides, with values of 6711.68 mm³ ± 796.61, and 8278.29 mm³ ± 1262.03, respectively.

**Table 3:** Comparison of different parameters between impaction and non-impaction sides in the PUMCI group

	Impaction side	Non-impaction side	Difference (95% CI)	*P* value
	Mean (SD)			
BD (GSV)	806.45 (43.40)	664.16 (42.86)	142.29 (134.43, 150.14)	< 0.001*
FD	1.21 (0.07)	0.91 (0.10)	0.30 (0.25, 0.34)	< 0.001*
MBV (mm³)	6688.29 (906.26)	5303.08 (1312.89)	1385.21 (1049.66, 1720.77)	< 0.001*
PV (mm³)	6711.68 (796.61)	8278.29 (1262.03)	-1566.61 (-1936.87, -1196.36)	< 0.001*

SD: Standard Deviation, CI: Confidence Interval

Paired samples t-test was used

*Statistically significant at p-value < 0.05

### Comparison between the impaction sides in both study groups

Upon comparing all the reported outcomes between the impaction sides in both groups, palatal impactions showed significantly greater values in contrast to buccal impactions as displayed in Table 4, for the following variables: MBV (*p* = 0.03), BD and FD (*p* < 0.001). Conversely, sides involving buccal impactions exhibited significantly greater PVs when compared to those measured on the palatal impaction sides (*p* = 0.02).

**Table 4:** Comparison of different parameters between BUMCI and PUMCI (impaction sides only)

	BUMCI	PUMCI	Difference (95% CI)	*P* value
	Mean (SD)			
BD (GSV)	672.25 (82.28)	806.45 (43.40)	-134.20 (-184.12, -84.28)	< 0.001*
FD	1.11 (0.04)	1.21 (0.07)	-0.10 (-0.14, -0.05)	< 0.001*
MBV (mm³)	5714.74 (1432.29)	6688.29 (906.26)	-973.55 (-1869399, -77.11)	0.03*
PV (mm³)	7767.62 (1410.84)	6711.6833 (796.61)	1055.94 (188.60, 1923.27)	0.02*

SD: Standard Deviation, CI: Confidence Interval

Independent samples t-test was used

*Statistically significant at p-value < 0.05

## Discussion

With unilateral maxillary canine impaction whether buccal or palatal in position considered an asymmetric anomaly of the maxillary arch, understanding the underlying skeletal characteristics accompanying this commonly encountered abnormality is of prime importance. Thus, the objective of the present study was to undergo a comprehensive skeletal assessment in unilateral canine impaction cases, both the buccally and the palatally positioned, including bone density, bone microstructure in terms of fractal dimension, bone volume, and palatal volume. As per the obtained results, the null hypothesis has been rejected, where statistically significant differences have been observed in the assessed skeletal factors between the impaction and the non-impaction sides in both positions, except for the PV in buccally impacted cases.

Conforming to the recommendations of the American Academy of Oral and Maxillofacial Radiology [31], CBCT scans were used in the identification of the dental and skeletal anomalies accompanying canine impaction. Accordingly, the acquired CBCTs were originally obtained to help in the accurate diagnosis and localization of the canines in question, in order to plan the most suitable treatment approach. CBCTs also offer the advantages of less radiation exposure, lower cost, and superior image accuracy in contrast to traditional computed tomography (CT) scans [32]. On another note, image blurring, overlapping structures and superimpositions that are experienced with panoramic x-rays, are all downsides that are eliminated with CBCT use [32]. Reviewing the literature, CBCTs have consistently been the preferred method employed in the diagnosis of canine impactions [33], as well as in the assessment of skeletal changes [7, 19, 34]. Bjerklin and Ericson [33] stated that the supplementary data given from CBCT scans resulted in treatment plan alterations in approximately 43.7% of their patients.

In the present study, bone density GSV have been found to be significantly greater on the buccal as well as on the palatal canine impaction sides, in comparison to the non-impaction sides, with the greatest BD values belonging to palatal impaction. Park et al. [35] have stated previously that the highest maxillary alveolar and basal bone density is located in the canine area, while investigating the best mini-screws’ placement sites. More specifically, in canine impaction cases, Sharhan et al. [32] reported higher BD in conjunction with canine impactions, whether unilateral or bilateral in comparison with the controls, supporting our study outcomes. The same findings have also been confirmed by Seçgin et al. [9] However, Al-Tawachi et al. [19], upon comparing BD on unilateral buccal and palatal canine impaction sides with their contra-laterals, they reported that the greatest BD values were attributed to palatal impactions in accordance with our reported outcomes, but the least BD was reported to be on the buccal impaction sides, a finding that contradicts our results where BD on the sides of buccal impactions were also significantly greater than those of the controls or the non-impaction sides.

Owing to the potential unreliability of GSV in the radiographic calculation of BD due to factors that include relatively high radiation scatter, restricted field size, and constraints of reconstruction algorithms [19], the reported GSV and their interpretation in terms of BD values should be illustrated with caution, and further affirmation might be required to advocate these findings. Therefore, assessment of bone microstructure in the form of FD has been performed to confirm the reported findings and to reduce the impact of any potential confounding factors. The selected ROI for FD analysis was chosen in a region of best fit in the maxillary alveolar process; between the first and second premolars on both the tested sides. Owing to the variability in the impacted maxillary canine position, the adjacent premolar region was the closest reproducible measurement area, to attain consistent trabecular images devoid of overlapping anatomical structures^(6)^.

Fractal dimension analysis in the present study revealed a similar pattern to that observed with BD, with the impaction sides in both BUMCI and PUMCI groups showing higher FD than the non-impaction sides, with the greatest FD being attributed to the sides with palatal canine impactions. Denser bone microstructure in association with both buccally and palatally impacted canines has also been pointed out by Arvind et al. [7], where they considered it as a possible etiologic factor contributing to canine impaction regardless of its direction. On the contrary, Servais et al. [6] reported non-significant FD differences between both sides in unilateral canine impaction cases.

For MBV, significantly greater volumes have been documented on the non-impaction sides in BUMCI group, with the opposite being reported in the PUMCI group, where greater MBVs have been calculated on the impaction sides when compared to the non-impaction sides. Therefore, buccal canine displacement tends to correlate with reduced MBV due to less bone support in the buccal region compared to the palatal region. This nuanced understanding highlights the biomechanical implications of impaction position on bone structure. An analogous observation was also reported by Al-Tawachi et al. [19]. These outcomes support the commonly reported concurrence of buccal canine displacement with deficiencies of the dental arch [36], and palatal canine displacements with sufficient arch space [37].

Another assessed outcome in the current study was the PV. Non-significant differences in PV have been calculated in the BUMCI group between both the tested sides, whereas in the PUMCI group, statistically greater PV has been noted on the non-impaction sides in contrast to the impaction sides. This is in accordance with the study by Yassaei et al. [20], which is the most recent investigation assessing palatal volume in cases with unilateral palatal canine impactions, and they reported a significant correlation between palatal canine impaction and palatal volume.

From a clinical perspective, understanding these skeletal variations can significantly inform treatment decisions. For instance, elevated BD in impaction sites can suggest a need for more robust orthodontic appliances and possibly prolonged treatment durations to effectively manage the impacted canines. Additionally, knowledge of higher FD values, particularly in palatally impacted canines, can alert clinicians to the potential for increased resistance during orthodontic tooth movement, thereby necessitating adjunctive surgical procedures or more frequent monitoring to ensure effective treatment progression.

Furthermore, the assessment of MBV and PV can aid in treatment planning by providing insights into the spatial dynamics of the maxillary arch. For example, reduced MBV on the buccal impaction side may indicate a need for arch expansion techniques, while greater MBV on the palatal impaction side suggests adequate space that may not require extensive arch modifications. Understanding PV variations, especially with palatally impacted canines, can also help in anticipating and managing potential complications related to palatal growth and development, ensuring that interventions are appropriately timed to coincide with the patient’s growth patterns.

A limitation of the current study is the relatively small sample size, which goes back to the retrospective nature of the study, in addition to the strict eligibility criteria implemented to create a homogenous sample. Future research with larger, more diverse samples will be essential to validate these findings and enhance their generalizability, advancing the field of orthodontics and contributing to a more personalized approach to managing unilateral maxillary canine impaction. It is also noteworthy to mention that since the same CBCT device was used for all the included scans, the GSV calculated for evaluation of BD could not be applied to those calculated by other devices pertaining to the differences in the image acquisition parameters.

## Conclusions

As per the reported results in the present study, the following conclusions have been drawn in unilateral canine impaction cases:

1. Buccal canine impactions are accompanied with significantly greater BD and FD, and significantly less MBV, in comparison to the non-impaction sides. PV differences were non-significant between both sides.
2. Palatally impacted canines are associated with significantly greater BD, FD, and MBV, in addition to significantly less PV, in contrast to the non-impaction sides.
3. Comparison of the measured outcomes between the buccal and palatal impaction sides showed significantly greater values for BD, FD, and MBV with palatally displaced canines, whereas with PV, significantly less volumes have been observed with palatal impactions in comparison with buccally displaced canines.
4. The findings suggest that higher BD and FD values, particularly in palatal impactions, could contribute to the difficulty of orthodontic interventions, necessitating more robust appliances, extended treatment durations, and possible adjunctive surgical procedures. Additionally, the assessment of BV and PV aids in refining treatment plans by providing a clearer understanding of the maxillary arch’s spatial dynamics, allowing for more precise interventions tailored to each patient’s unique skeletal structure.

## Data Availability

The datasets used and/or analyzed during the current study are available from the corresponding author on reasonable request.

## Abbreviations

CBCT: Cone-beam computed tomography
BD: Bone density
GSV: Grey scale values
FD: Fractal dimension
BV: Bone volume
PV: Palatal volume
3D: Three-dimensional
SD: Standard deviation
BUMCI: Buccal Unilateral Maxillary Canine Impaction
PUMCI: Palatal Unilateral Maxillary Canine Impaction
FOV: Field of view
ROI: Region of interest
MBV: Maxillary bone volume
STL: Standard Tessellation Language
ANS: Anterior nasal spine
CEJ: Cemento-enamel junction
CI: Confidence interval
ICC: Intraclass correlation coefficient
